# Evaluation of Genomic Surveillance of SARS-CoV-2 Virus Isolates and Comparison of Mutational Spectrum of Variants in Bangladesh

**DOI:** 10.3390/v17020182

**Published:** 2025-01-27

**Authors:** Abeda Sultana, Laila Anjuman Banu, Mahmud Hossain, Nahid Azmin, Nurun Nahar Nila, Sharadindu Kanti Sinha, Zahid Hassan

**Affiliations:** 1 Department of Anatomy, Dhaka Medical College, Dhaka 1000, Bangladesh; abedabsmmu@gmail.com; 2 Genetics and Molecular Biology Laboratory, Bangabandhu Sheikh Mujib Medical University, Dhaka 1000, Bangladesh; 3 Laboratory of Neuroscience and Neurogenetics, Department of Biochemistry and Molecular Biology, University of Dhaka, Dhaka 1000, Bangladesh; mahmudbio1480@du.ac.bd (M.H.); nurunnahar-2015517630@bmb.du.ac.bd (N.N.N.); 4 Department of Anatomy, Shahabuddin Medical College, Dhaka 1212, Bangladesh; nahidazmin@gmail.com; 5 Department of Pharmacology, Bangabandhu Sheikh Mujib Medical University, Dhaka 1000, Bangladesh; sharadindu@bsmmu.edu.bd; 6 Department of Physiology and Molecular Biology, Bangladesh University of Health Sciences, Dhaka 1216, Bangladesh; mzhassan@buhs.ac.bd

**Keywords:** SARS-CoV-2, COVID-19, Omicron, Delta, Mauritius, mutation, Bangladesh

## Abstract

The SARS-CoV-2-induced disease, COVID-19, remains a worldwide public health concern due to its high rate of transmission, even in vaccinated and previously infected people. In the endemic state, it continues to cause significant pathology. To elu- cidate the viral mutational changes and screen the emergence of new variants of concern, we conducted this study in Bangladesh. The viral RNA genomes extracted from 25 ran- domly collected samples of COVID-19-positive patients from March 2021 to February 2022 were sequenced using Illumina COVID Seq protocol and genomic data processing, as well as evaluations performed in DRAGEN COVID Lineage software. In this study, the percentage of Delta, Omicron, and Mauritius variants identified were 88%, 8%, and 4%, respectively. All of the 25 samples had 23,403 A>G (D614G, S gene), 3037 C>T (nsp3), and 14,408 C>T (nsp12) mutations, where 23,403 A>G was responsible for increased transmis- sion. Omicron had the highest number of unique mutations in the spike protein (i.e., sub- stitutions, deletions, and insertions), which may explain its higher transmissibility and immune-evading ability than Delta. A total of 779 mutations were identified, where 691 substitutions, 85 deletions, and 3 insertion mutations were observed. To sum up, our study will enrich the genomic database of SARS-CoV-2, aiding in treatment strategies along with understanding the virus’s preferences in both mutation type and mutation site for predicting newly emerged viruses’ survival strategies and thus for preparing to coun- teract them.

## 1. Introduction

The novel Severe Acute Respiratory Syndrome Coronavirus 2 (SARS-CoV-2), which introduced itself as a global health threat, was first reported on 30 December 2019 in Wuhan city of Hubei province in China [1], and its associated disease is known as Coronavirus Disease 2019 (COVID-19) [2]. The devastating accelerated geographic spread of COVID-19 resembles the SARS and Middle East Respiratory Syndrome (MERS) outbreaks, which also resulted in pandemic situations [3,4]. According to the World Health Organization (WHO), the total number of infected individuals is above 776 million, and the death count is beyond 7 million. In Bangladesh, the first three COVID-19 cases were reported officially on 8th March 2020, having a travel history from Italy, where COVID-19 had already spread rapidly [5]. In Bangladesh, 2,051,201 people were infected with SARS-CoV-2, and over 29,000 people died from COVID-19, as per information provided by the government-designated institution [6].

As a spherical pleomorphic viral particle, coronavirus possesses the longest non-segmented RNA genome of 26 to 32 kb in length, which is also positive-sense single-stranded in nature with a 5′ cap and 3′ poly-adenylation [7,8,9,10]. The genome includes 14 functional open reading frames (ORFs) comprising genes for 16 non-structural (nsp1-nsp16), 4 structural, and 9 accessory proteins, along with two non-coding regions at both ends [11,12]. ORF1a and ORF1b encode 16 NSPs that are essential for viral RNA synthesis, while accessory proteins provide selective facilities in the host cell for its pathogenicity [13,14]. This virus is made of four types of structural proteins that are vital for viral assembly, i.e., spike (S), membrane (M), envelope (E), and nucleocapsid proteins (N), and among them, the spike protein is the most significant one for attachment, fusion, and entry into the host cell [15,16]. The S, M, and E proteins are embedded in the envelope, and the N protein forms the core structure. The spike protein is a trimeric protein, where its monomer comprises two distinct functional subunits, S1 and S2. These subunits are necessary for attachment and membrane fusion, respectively, which lead to its entrance into the host cell [17]. The viral genome is encapsulated with the nucleocapsid protein that forms a helical ribonucleoprotein (RNP) complex [18]. Moreover, envelope and membrane proteins form the envelope and the membrane of the virus, respectively, and thus the membrane proteins give a distinct shape to the virus [19,20].

COVID-19, which emerged through a zoonotic transmission event [21], is an acute disease that is persistent in immune-suppressed individuals [22,23]. Viral infection occurs due to human-to-human transmission by being in contact with the droplets of saliva that come out while talking, coughing, and sneezing from a close distance or due to direct contact with oral, nasal, and eye mucous membranes [24,25,26]. The incubation period of the SARS-CoV-2 virus ranges from 2 to 14 days, and the symptoms are similar to those of influenza with some other clinical manifestations, i.e., fever, dry cough, myalgia, headache, nausea or vomiting, chills, diarrhea, conjunctival congestion, shortness of breath, sore throat, and fatigue [27,28,29]. Being infected with SARS-CoV-2, the mortality rate is observed to increase with age, where the rate increases significantly above 80 years of age, and the other comorbidities that are believed to play a role in the increasing mortality rate are heart disease, diabetes, chronic lung or kidney disease, and other socio-demographic factors. Moreover, an increased risk of being infected is observed to be associated with hypertension, diabetes, and coronary heart disease [30,31]. However, previous infection with COVID-19 and vaccination are the protective factors against SARS-CoV-2 virus infection [32]. For rapid diagnosis, Real Time-Polymerase Chain Reaction (RT-PCR) and Reverse Transcriptase-Loop Mediated Isothermal Amplification (RT-LAMP) techniques are used to detect viral RNA in the laboratory [33].

After the first wave of COVID-19, several variants were observed when compared to the reference genome of SARS-CoV-2 (GenBank). Viruses generally acquire an alteration of their genetic constitution over time, giving rise to new variants, more specifically, said variants of interest (VOI) or variants of concern (VOC), which usually have increased transmissibility and ability to escape immunity and/or have severe pathogenicity. Concerning variants are often referred to as variant, strain, and lineage interchangeably or by the country in which they were first identified. The term “emerging variant” is used to label a new variant that appears in a population [34]. According to the WHO, the reported COVID-19 cases are decreasing in Greece, Northern Ireland, Poland, New Zealand, and the United Kingdom of Great Britain. In addition, at present, according to the “COVID-19 Dynamic Dashboard for Bangladesh” from the website of the Directorate General of Health Services (DGHS), Bangladesh (accessed on 16th December 2024), the confirmed COVID-19 cases are at the lowest point, and in the last 24 h, 2 patients were confirmed to be COVID-19-positive.

Vaccination is the key to controlling SARS-CoV-2 infection. Evidence proves that the risk of death, hospitalization, mild/moderate/severe disease, and incidents of infection is reduced due to the administration of different types of vaccines [35,36]. However, a big threat is posed when VOCs emerge, which challenge the effectiveness of vaccines, and this was evident in the incidence of Pfizer-BioNTech and AstraZeneca vaccines being less effective against the beta variant compared to the wild type. The new mutant strains of SARS-CoV-2, with greater infecting ability and immune-evading characteristics, led to new waves of COVID-19 infection and death. Till now, the Alpha, Beta, Gamma, Delta, and Omicron variants emerged with changes in viral behavior, infectivity, and pathogenicity, i.e., the ability to evade the immune system by altering their Receptor Binding Domain of the S protein [37]. For this reason, regular surveillance of VOCs via whole-genome sequencing is required to inform global vaccination programs [38]. Hence, every day, many datasets of SARS-CoV-2-virus’s whole-genome sequences are being deposited in genomic databases from different parts of the world. However, the number is very low in the case of the SARS-CoV-2 virus that caused great havoc in Bangladesh. Keeping in mind the necessity of the surveillance of new VOCs, we sequenced the genome of SARS-CoV-2 from 25 COVID-19-positive patients via Next Generation Sequencing (NGS) technology and evaluated their clade, lineage, signature mutation, and mutational spectra.

## 2. Materials and Methods

### 2.1. Study Subject Selection

The Institutional Review Board (IRB) of Bangabandhu Sheikh Mujib Medical University (BSMMU), under the Declaration of Helsinki’s ethical principles, provided formal approval (No.BSMMU/2021/5961, date 28 June 2021, Reg. No. 3506) to conduct the present study. This cross-sectional descriptive study was conducted at the Genomic Research Laboratory at the Department of Anatomy, BSMMU, Bangladesh, from March 2021 to February 2022. For collection of specimens, a consortium was formed with the Laboratory of Neuroscience and Neurogenetics, Department of Biochemistry and Molecular Biology, University of Dhaka, Bangladesh; Department of Physiology and Molecular Biology, Bangladesh University of Health Sciences, Dhaka, Bangladesh; The National Institute of Laboratory Medicine & Referral Center (NILMRC), Dhaka, Bangladesh; and Dhaka Medical College, Dhaka, Bangladesh. After having informed written consent, 25 COVID-19-positive patients who met the inclusion and exclusion criteria were selected. Using the patients’ selection checklist and data collection questionnaire, the socio-demographic and comorbidity data of the patients were collected.

### 2.2. Ethical Implication

In this research, all the patients were treated equally and with due respect. They were informed about the aim and possible benefits of the variants’ characterization before asking for their written consent in a prescribed consent form. It was also made clear to the patients that their RNA would only be used for research purposes, they could withdraw their name anytime during the study, and any withdrawal would not affect their treatment. Moreover, to avoid duplication and to ensure safeguarding confidentiality, each of the COVID-19 patients was given a unique ID number.

### 2.3. Isolation of RNA

Genomic RNA was extracted from nasopharyngeal swab samples of selected COVID-19 patients using ReliaPrep™ (Promega, Madison, WI, USA) RNA extraction kit. Micro-centrifuge tube with 200 μL nasopharyngeal swab was treated sequentially with the enzyme Proteinase K (Promega, Madison, WI, USA), cell lysis buffer (Promega, Madison, WI, USA), isopropanol (Merck, Darmstadt, Germany), and wash buffer (Illumina, San Diego, CA, USA), where Proteinase K and cell-lysis buffer were used for cell lysis and denaturation of proteins, i.e., nuclease as well as other proteins, and to assist in removal of these pro- teins from the RNA extraction specimen following centrifugation. In addition, isopropa- nol was used for precipitation of RNA that forms a gel-like pellet.

### 2.4. Synthesis and Amplification of cDNA

Nanodrop 2000/2000c spectrophotometer (Thermo Fisher Scientific Inc., Waltham, MA, USA) was used for concentration measurement and to check the quality of viral genomic RNA. Using ran- dom sequence hexamers, the isolated RNAs were annealed. Afterward, reverse transcrip- tase (Promega, Madison, WI, USA) was used to reversely transcribe these RNA fragments into their complementary DNA. Thus, the first strand of cDNA was synthesized. cDNA amplification was performed using two sets of COVID Seq primers in 2 separate PCR re- actions along with these reagents—IPM HT (Illumina PCR Mix HT, Illumina, San Diego, CA, USA), CPP1 HT (COVID Seq Primer Pool 1HT, Illumina, USA), and CPP2 HT (COVID Seq Primer Pool 2HT, Illumina, USA).

### 2.5. Amplification of Tagmented Amplicons and Library Preparation

Tagmentation of PCR amplicons with adapter sequence was facilitated by using EBLTS-HT (Enrichment BLT HT, Illumina, USA) and TB1 HT (Tagmentation Buffer 1 HT, Illumina, USA). This step executes fragmentation as well as tagging of PCR amplicons with adapter sequences. In post-tagmentation clean up step, adapter-tagged amplicons were washed with ST2 HT (Stop Tagment Buffer 2 HT, Illumina, USA) and TWB HT (Tagmentation Wash Buffer HT, Illumina, USA) before performing their PCR amplification. The tagmented amplicons were amplified using a PCR program that includes EPM HT (Enhanced PCR Mix HT, Illumina, USA), index adapters, and sequences that were required for sequencing clues. Libraries from all the 96-well plates were then combined and placed in a 1.7 mL tube, followed by binding of optimal size libraries to the magnetic bead using ITB (Illumina Tune Beads, Illumina, USA). Afterward, RSB (Resuspension Buffer HT, Illumina, USA) washed away the too-large or small fragments. Thus, pooling and cleaning of libraries were accomplished. Qubit dsDNA HS Assay kit (Thermo Fisher, Waltham, MA, USA) was employed for analysis of 2 µL library pool. Standard range of libraries were maintained, and RSB was used for 10× dilution and then analyzed once again. The average library size was 400 bp, and the diluted normalized concentration was 4 nM. As per library preparation documentation, for denaturation and dilution, library’s standard normalization method was applied. Libraries were diluted to the starting concentration (according to the manufacturer’s guidelines) for the sequencing system.

### 2.6. Loading of the Libraries to the MiSeq Flow Cell and Sequencer Run

The denatured and diluted libraries were loaded into the reagent cartridge, and then the sequencing run was set up. For evaluation of sequence reads of RNA libraries, the Illumina DRAGEN COVID Seq Test Pipeline was used. First, the sequence option was selected from the screen of the software interface, which was followed by the selection of the run setup option. The reagent cartridge was loaded, the run parameters were reviewed, and results were checked via pre-run. After selection of the Start button, run was observed using either Local Run Manager or Sequencing Analysis Viewer (SAV) from the MCS interface. Finally, the libraries were loaded to the MiSeq flow cell, and the system was run. After running, data were automatically uploaded into BaseSpace Sequence hub by base calling within 12 to 13 h. FASTQ files were uploaded and analyzed in DRAGEN COVID Lineage software (version 3.5.9), which helps in assembling SNV with reference SARS-CoV-2 genome. One Excel and other FASTA files were generated in BaseSpace, whereas the Excel file included clade and lineage files. The SARS-CoV-2 variant evaluation was performed by using COVID-Seq Lineage software (version 3.5.9).

### 2.7. Sequence Data Processing and Evaluation

The raw output was the BCL formatted file, which was eventually converted into FASTA format after several steps. To detect viral pathogen coverage, k-mer-based approaches were exploited. Reads were aligned to a SARS-CoV-2 reference genome, and a consensus genome sequence was generated. Lineage/clade analysis was performed using Pangolin. DRAGEN RNA Pathogen Detection Pipeline encompasses human and viral references, which is a combination of reference sequences of the human genome (hg38) with selected reference sequences of the virus. Moreover, this pipeline consists of an integrated FASTA generation feature that can be uploaded to public databases, i.e., in our case, the GenBank Database. The accession numbers of the 25 virus genomes are OM019149, OM019148, OM019138, OM019145, OM019139, OM019150, OM019143, OM019146, OM090130, OM090137, OM090136, OM090135, OM090140, OM019153, OM019152, OM019154, OM019140, OM019147, OM019141, OM019155, OM090139, OM277491, OM277497, OM277498, and OM019151. The resulting sequences of our study were compared with the GenBank of National Centre for Biotechnology Information (NCBI) database. Furthermore, MEGA X software (version 10.5) automatically translated the codons. Thereafter, the NCBI Entrez search engine aided in the comparison of translated codons with the reference sequence.

### 2.8. Statistical Analysis and Graph Construction

Statistical Package for Social Sciences ((SPSS), version 23) was used for statistical and mutational analysis, i.e., determining the mutation count and percentage of specific mutation (i.e., D614G) falling in a specific category of mutation (i.e., substitution mutation) observed in the viral genome from COVID-19 patients and for the data of socio-demographic and disease condition-related characteristics. In this study, pie charts and lollipop plots were constructed for better visualization of the data using R studio (Version: 2024.12.0+467) and the updated version of SRPlot (SRplot- Science and Research online plot), respectively [39]. 

## 3. Results

### 3.1. Socio-Demographic and Disease-Related Data of COVID-19 Patients

Among the twenty-five adult SARS-CoV-2 positive patients, 48% were males and 52% were females. Most of them (84%) had no family history of COVID-19. The quarantine period was maintained by 48% of the patients. Twelve percent (12%) had comorbidities like hypertension, but only four percent had a history of hypertension with asthma. A history of other comorbidities like chronic kidney disease, diabetes mellitus, and chronic obstructive pulmonary disease conditions was absent. Substantially, only 12% of the patients had a second positive history of COVID-19 (Table 1).

**Figure 1:**
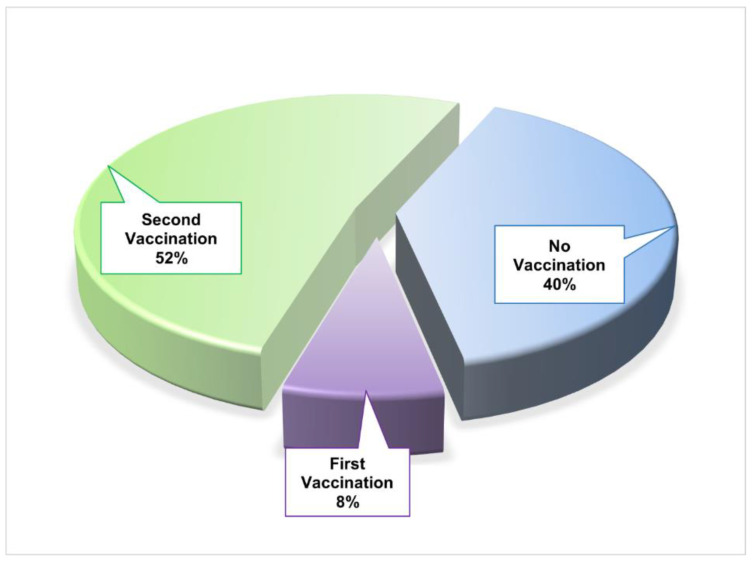
Vaccination status of COVID-19 patients.

Additionally, 4% of the patients remained positive for a long duration. Most of the people had no specific long traveling history. They moved only short distances, such as from home to the workplace, and some people had a history of social gatherings, such as invitations or religious festivals (Table 1). Fifty-two percent of the patients were vaccinated with two doses, while eight percent had taken only the first dose of the COVID-19 vaccine. The rest of the patients (forty percent) did not have any vaccine against SARS-CoV-2 (Figure 1).

### 3.2. SARS-CoV-2 Variants, Clades, and Lineage Evaluation

**Figure 2:**
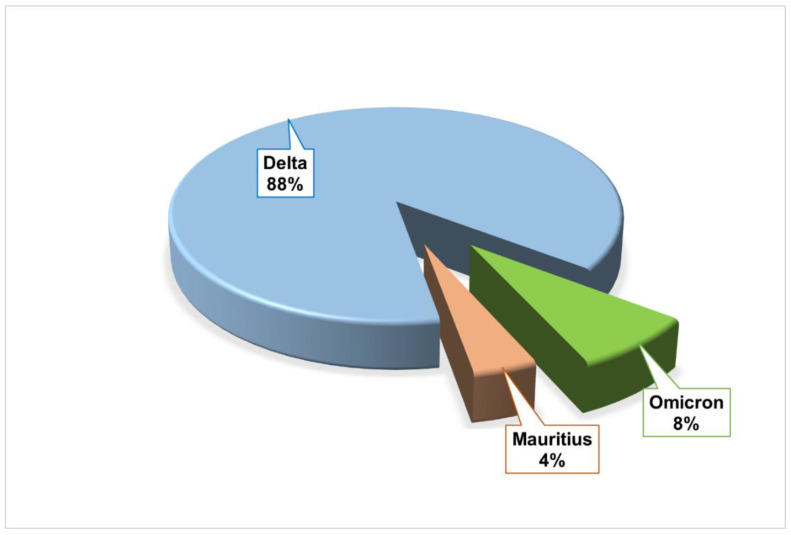
Distribution of SARS-CoV-2 variants in COVID-19 patients.

Omicron, Delta, and Mauritius variants were detected in the present study. Among the twenty-five COVID-19 patients, twenty-two were Delta variant carriers, two were Omicron carriers, and only one patient was identified to be infected with the Mauritius variant of SARS-CoV-2 (Figure 2).

Evaluation of the viral clades demonstrated that the 21A clade and 21J clade of the Delta variants comprised 80% and 8% of all the variants, respectively. In addition, the 20B clade of the Mauritius variant was found in four percent of the patients, whereas the 20A clade and 21K clade of the Omicron variants were found in the remaining 8% of patients (Table 2).

Lineage analysis of the SARS-CoV-2 variants revealed that in the case of Delta variants, 28% were under the B.1.617.2 lineage, 24% were under the AY.4 lineage, 8% were under the AY.131 lineage, 8% were under the AY.26 lineage, and the rest of the Delta variants came from a different category of lineages, like AY.122, AY.122.1, AY.29, AY.30, and AY.39 (Table 3). The Mauritius variant fell under B.1.1.318 (4%), while the two Omicron variants were under the BA.1 lineage (8%) (Table 3).

### 3.3. Substitution and Insertion–Deletion Mutation Analysis

A study on different types of mutations in three of the SARS-CoV-2 variants unveiled that in Omicron variants, the number of maximum and minimum substitutions were 53 (the highest among all three variants) and 41, respectively, while the maximum and minimum number of deletion loci were observed to be 6 and 6, with their maximum created gaps of 45 bp and 39 bp, accordingly (Table 4). In addition, two insertion mutations with a 9 nucleotide base length were identified in two Omicron variants.

However, Delta variants, on the other hand, exhibited maximum and minimum substitutions of 45 and 14, respectively. Meanwhile, for deletion mutations, the number of deletion loci were 4 and 3, with maximum created gaps of 18 bp and 13 bp, respectively. Noticeably, no insertion mutation was seen in the Delta variants (Table 4). Moreover, in the case of the Mauritius variant, the number of substitutions, deletion, and insertion mutations were 36, 5 (35 bp gap), and 1 (3 bp in length), respectively. Using Next Generation Sequencing of the whole viral genome of the extracted RNA of twenty-five COVID-19 patients, a total of 779 mutations were identified, of which 691 substitution, 85 deletion, and 3 insertion mutations were observed.

### 3.4. Comprehensive Investigation of Nucleotide Substitution Mutations

The most common substitution mutations were 23,403 A>G (S gene), 3037 C>T (ORF1a (nsp3) gene), and 14,408 C>T (ORF1b (nsp12) gene), of which mutations were present in all of the twenty-five samples (Table 5), whereas substitution mutation 26,767 T>C (M gene) was found in 23 samples, and 15,451 G>A (ORF1b (nsp12)), 25,469 C>T (ORF3a), and 23,604 C>G (S gene) were identified in 22 viral genomes.

These aforementioned mutations were detected in 21 (88%) samples, and the other mutations encompass the 5′ Leader Sequence, ORF1a, ORF1b, S, N, ORF7b, ORF7a, and the 3′ end region (Table 5). Mutations that were detected in at least four samples are shown in Table 5, and other minimal count mutations are given in Supplementary Table S1.

The most common type of alteration of the base pair was C>T, comprising around 38.48% of all the variants, which was followed by G>T (18.23%), A>G (11.52%), G>A (8.61%), and T>C (8.39%) in order. All of these aforementioned base changes are transition mutations, except for G>T (Table 6).

A comparison of the three variants’ viral genomes unveiled that in all three variants, the transition of nucleotide bases was way higher than the transversion mutation, and the percentages of transition and transversion mutation were 67% and 33%, respectively (Table 7).

### 3.5. Extensive Analysis of Nucleotide Deletion Mutations

An in-depth analysis of deletion mutations revealed that 22,029–22,035 bp (S), 28,248–28,254 bp (ORF8), 28,274 bp (N), and 29,750–29,752 bp (non-coding) deletions were identified in 20 Delta variant samples of the 21A clade, and the last one was observed in only 1 sample (Table 8).

Moreover, deletion in 22,029–22,034 bp (S gene), 28,248–28,253 bp (ORF8), and 28,271 bp (non-coding region) regions was noted in both 21J Delta variants samples except the 21,992–21,994 bp deletion mutation, which was found only in one sample. Additionally, most of the Delta variants had 15 bp deletions. On the contrary, in the case of Omicron variants, most of the deletions were unique, and these were different from Delta or Mauritius variants, i.e., deletion at 6513–6516/6513–6515 bp (ORF1a) was only observed in the Omicron variant and deletion at around the 11,287 bp (ORF1a) locus was noticed in both Omicron and Mauritius variants but not in the Delta variant. The two Omicron had 45 bp (6513–6516 (ORF1a), 11,287–11,296 (ORF1a), 21,766–21,772 (S), 21,987–21,996 (S), 22,194–22,197 (S), and 28,363–28,372 (N)) and 39 bp (6513–6515 (ORF1a), 11,285–11,293 (ORF1a), 21,765–21,770 (S), 21,987–21,995 (S), 22,194–22,196 (S), and 28,362–28,370 (N)) deletions at six deletion loci. However, 35 bp deletions at five deletion sites (11,288–11,297 (ORF1a), 21,994–21,997 (S), 27,887–27,902 (ORF7b, ORF8), 28,254 (ORF8), and 28,896–28,899 (N)) were revealed in the Mauritius variant, which also had unique deletions at 27,887–27,902 bp covering both ORF7b and ORF8 genes (Table 8).

### 3.6. Detailed Evaluation of Insertion Mutations

Two insertion mutations of 9 bp length in the spike glycoprotein region were seen in two of the Omicron variants, and they are 22,204: GAGCCAGAA and 22,206: GCCAGAAGA (Table 9). In the case of the Mauritius variant, an insertion mutation of 3 bp (28,250: CTG) in length was observed at the ORF8 region, and no insertion mutation was seen in the case of the Delta variant.

### 3.7. Missense Mutations Analysis in SARS-CoV-2 Viral Genome

A comparison of MEGA X translated codons with reference genomes facilitated the unraveling of amino acid substitutions. The amino acid substitution (missense/nonsense) mutations were obtained after analysis and were found in at least three viral genomic sequences (Table 10).

On the contrary, mutations that were least common and found in fewer than three samples are shown in Supplementary Table S2. The S: D614G missense mutation was the most frequent one and was present in all of the study subjects. Then, M: I82T was the most frequent in order, being present in 24 samples, and ORF3a:S26L, S:P681R, and ORF1b:G662S were the third most frequent mutations, being present in 22 samples. Moreover, ORF1b:P314L, N:M1X, ORF9b:T60A, S:T19R, and S:E156G missense mutations were found in at least 20 samples. Other mutations are presented in Table 10.

**Figure 3:**
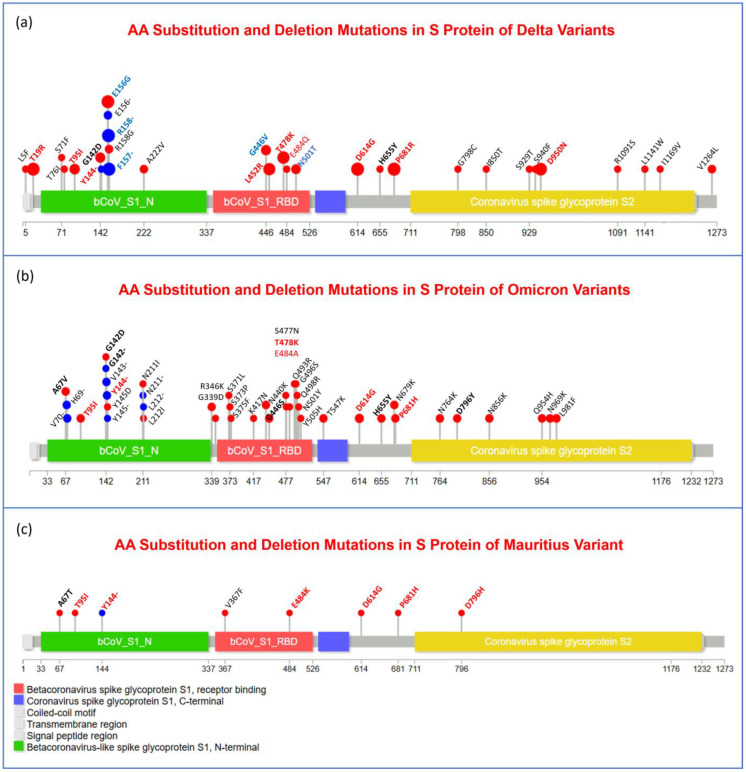
Needle plot of the visual representation of amino acid (AA) substitution (red lollipop) and deletion (blue lollipop) mutations in different domains and motifs of spike glycoprotein (S protein) of the Delta (**a**), Omicron (**b**), and Mauritius (**c**) variants of SARS-CoV-2 virus. Bold and blue font are used to refer to the most frequent mutations in a specific variant’s samples, while red color represents remarkable variants, and red-colored mutations are common for 3 variants. On the other hand, black and bold font represent common mutations for any of the 2 variants.

**Figure 4:**
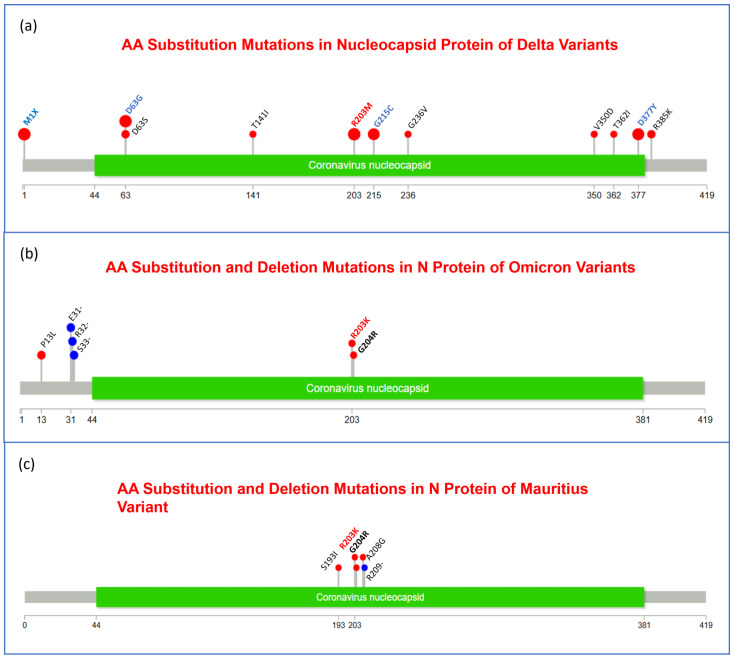
Lollipop plot representing the amino acid (AA) substitution and deletion mutations in nucleocapsid protein (N protein) of Delta (**a**), Omicron (**b**), and Mauritius (**c**) variants, where the red lollipop represents the aa substitution mutation, and deletion mutations are symbolized by the blue one. No deletion mutation was observed in the nucleocapsid protein of the Delta variant. Regarding the annotation of mutations, the bold red font represents the signature of important mutations reported in previous studies, while the bold blue font represents mutations that were found in a specific variant in highest frequency. Bold black font was used to refer to mutations that were present in 2 variants at least.

**Figure 5:**
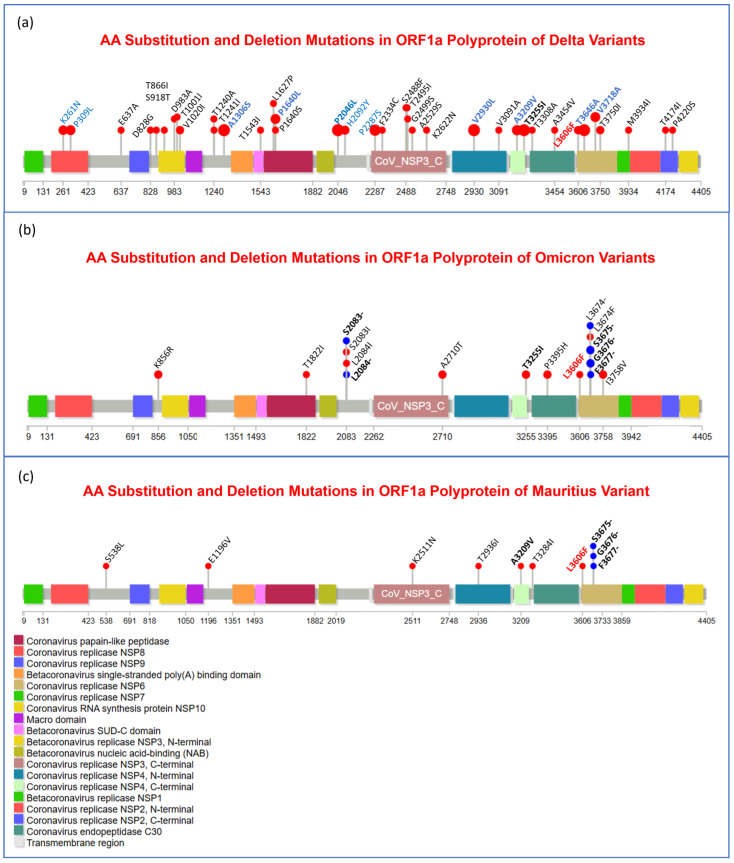
Substitution (red lollipop) and deletion (blue lollipop) mutations in ORF1a polyprotein region of the 3 variants, Delta (**a**), Omicron (**b**), and Mauritius (**c**), are shown in this needle plot. Annotation of mutations was performed based on the following font style—bold black font refers to mutations identified in at least 2 variants; bold blue font refers to mutations that were observed in highest frequency in a specific variant; bold red font indicates the noteworthy mutations and, in some cases, mutations that were found in 3 variants.

**Figure 6:**
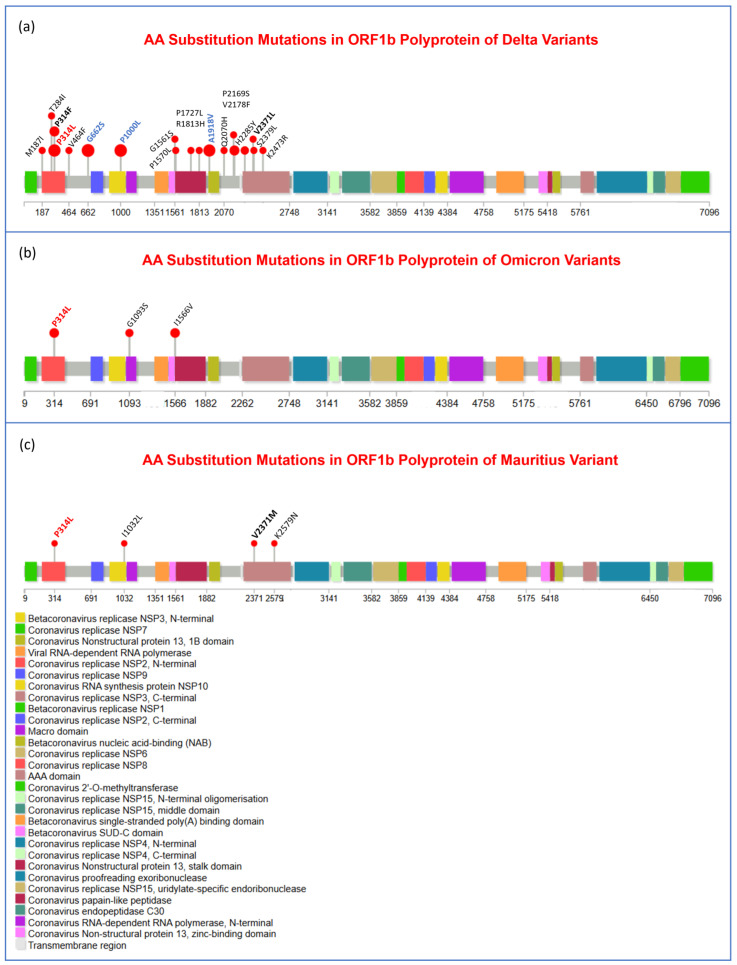
Exhibition of substitution mutations in ORF1b polyprotein region in Delta (**a**), Omicron (**b**), and Mauritius (**c**) viral genomes. No amino acid deletion was observed in this region of ORF1b gene. Here, the red lollipop presents the aa substitution mutation, and deletion mutations are symbolized by the blue one. For annotation of the mutations, bold black font refers to mutations that were present in 2 variants; the bold blue one represents mutations that were present in most of the samples of a specific variant; and bold red font implies the remarkable mutations and, in some cases, it refers to the mutations that were present in all 3 variants.

Moreover, to visualize the mutational spectra of S and N proteins and OR1a and ORF1b polyproteins, lollipop plots of amino acid substitution and deletion mutations in all three variants are given in Figure 3, Figure 4, Figure 5 and Figure 6 accordingly. The mutations that were observed in more than one variant are indicated in bold letters and black in color, and the mutations that play important roles in viral pathogenesis and transmission are indicated in bold font and red color.

Furthermore, mutations that were present in most of the samples of a specific variant are bolded, blue in color, and have larger lollipops. In all these figures (Figure 3, Figure 4, Figure 5 and Figure 6), panel (a), panel (b), and panel (c) represent Delta, Omicron, and Mauritius variants, respectively. In Figure 3, it is clear that in the spike protein of the Omicron variant, most of the unique mutations and deletion and substitution mutations were noticed. Substitution mutations T19R, E156G, L452R, T478K, D614G, P681R, and D950N were observed in most of the Delta variants, and T95I and D614G were found in all three variants. Moreover, P681 was substituted with R (Arginine) in Delta but with H (Histidine) in Omicron and Mauritius variants, while A67 was substituted with V (Valine) in Omicron, but it was substituted with T (Threonine) in the Mauritius variant. In addition, D796 was substituted with Y (Tyrosine) in the Omicron variant and with H in the Mauritius variant. Furthermore, G142D, T478K, and H655Y were found both in Delta and Omicron variants. Interestingly, E484 was substituted with Q, A, and K in Delta, Omicron, and Mauritius variants, respectively.

However, in the nucleocapsid protein (N) of the Delta variant, no amino acid deletion was observed, and compared to the other two variants, most of the mutations were in the Delta variant (Figure 4). Interestingly, only R203 was found to be substituted in all these three variants, but it was substituted with M (Methionine) in Delta, while it was substituted with K (Lysine) in Omicron and Mauritius variants. In the Omicron and Mauritius variants, the common mutation was G204R. Moreover, M1X, D63G, R203M, G215C, and D377Y were the most frequent mutations in Delta variant samples.

However, regarding ORF1a, in the case of Delta mutations, it encompassed the whole polyprotein region, and the other two variants showed fewer mutations in the N terminal regions (Figure 5). In Delta variants, K261N, P309L, A1306S, P1640L, H2092Y, P2287S, V2930L, A3209V, T3255I, T3646A, and V3718A were the most frequent mutations. Moreover, when comparing the variants, only L3606F was observed to be common in all three variants, and A3209V was observed in the Delta and Mauritius variants, whereas T3255I was found in both Delta and Omicron variants.

In our investigation, we found that the ORF1b protein did not have mutations at all after the 2473rd amino acid residue and downright from the N terminal domain of NSP4 (Figure 6). However, P314L was observed in all three variants, and in some samples of the Delta variant, P314 was substituted with F (Phenylalanine), while V2371 was substituted with L (Leu) in the Delta variant, which was substituted with M (Methionine) in the Mauritius variant. In addition, P314L, G662S, P1000L, and A1918V were the most frequent mutations in the Delta variant viral genomes.

### 3.8. Amino Acid Deletion Mutation Analysis

Deletion mutation analysis of the Omicron variant genome revealed that N:E31-, N:R32-, N:S33-, ORF1a:S3675-, ORF1a:G3676-, ORF9b:N28-, ORF9b:A29-, S:H69-, S:V70-, S:V143-, S:Y144-, ORF1a:S2083-, ORF1a:L2084-, ORF1a:L3674-, ORF1a:F3677-, ORF9b:E27-, ORF9b:V30-, S:G142-, S:Y145-, S:N211-, and S:L212- deletions occur in nucleocapsid, non-structural proteins, and spike protein-encoding regions (Table 11).

However, in the Mauritius variant, there were seven amino acid deletion mutations (N: R209-, ORF1a: S3675-, ORF1a:G3676-, ORF1a:F3677-, ORF8:M1-, ORF8:K2-, and S:Y144-), and they were located in 1 nucleocapsid, 5 non-structural proteins, and 1 spike protein-encoding region (Table 11). On the other side, six amino acid deletion mutations were observed in the spike protein and non-structural protein encoding regions of the Delta variant, and they are ORF8:D119-, ORF8:F120-, S: F156-, S: F157-, S:R158-, and S:Y144- (Table 11). Figure 3 suggests that the Y144- deletion mutation occurs in the S protein of all three variants. In the ORF1a protein, it was observed that S3675-, G3676-, and E3677- were common mutations for Omicron and Mauritius variants (Figure 5). In Figure 6, we noticed that in the ORF1b protein, none of the variants had a deletion mutation.

### 3.9. Proteins with the Highest Mutation Count

**Figure 7:**
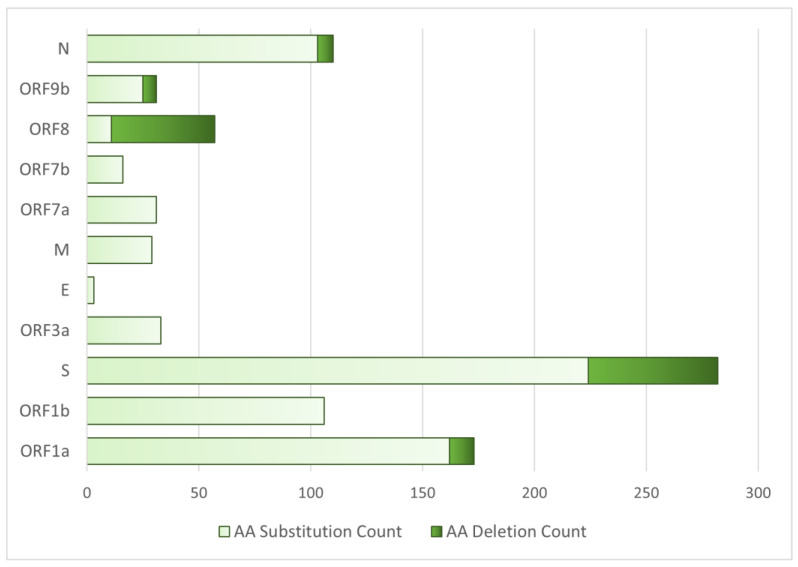
Amino acid (AA) substitution and AA deletion mutation numbers in different proteins of viral genomes.

The amino acid substitution and deletion mutation count in different proteins of the SARS-CoV-2 virus was observed in the 25 samples (Figure 7). Clearly, the figure states that in order, spike protein (S), ORF1a, and ORF1b and the Nucleocapsid (N) protein were highly mutated by substitution mutations. Moreover, ORF8 and S proteins have the highest amino acid deletion mutation count. On the other hand, none of the SARS-CoV-2 variants acquired deletion mutations at the ORF1b, ORF3a, E, M, ORF7a, and ORF7b gene regions.

## 4. Discussion

The rapid geographical spread of the novel coronavirus disease, COVID-19, that commenced at the end of 2019 left a pernicious and traumatic impact on every aspect of human life. It spread drastically and rapidly around the whole world, posing a global health threat that even now is prevailing and circulating in different regions of the world, taking away more lives [40]. The secret of this viral particle being uncontrollable altogether is believed to be hidden in its threatening potential of attaining genetic diversity due to random recombination and mutations of different categories like synonymous and non-synonymous mutations (substitution) and insertion–deletion mutations [41]. SARS-CoV-2 is an RNA virus that acquires mutations due to the occurrence of random nucleotide sequence error, which facilitates the virus in attaining genetic alterations that provide advantages in increasing its adaptability, pathogenicity, and transmissibility [40]. However, though the RNA virus acquired mutations because of the low fidelity of RNA-dependent RNA polymerase, it has 3′ to 5′ exonuclease activity that performs the proofreading activity, which explains the slower rate of mutation attainment of this viral particle [42].

Therefore, to combat this continual threat and havoc evoked by this virus, an analysis of genomic surveillance, mutational spectrum, viral behavior, and its related pathogenesis, as well as infectibility, is crucial for adopting preventive strategies like the development of therapeutics and vaccines of different categories [43,44]. Potential therapeutic strategies include the development of FDA-approved drugs; the use of RNA interference [45] as a weapon to combat SARS-CoV-2, i.e., siRNA-based therapeutics [46]; miRNAs as antiviral therapy [47]; and circRNAs as antiviral targets [48]. Moreover, recent startling techniques for vaccine development that are under evaluation include DNA/RNA vaccines and Epitope-based vaccines [49,50,51]. Recurrent mutations occur in RNA viruses, and SARS-CoV-2 is not different from this. Thus, designing and developing treatment therapeutics and vaccines for controlling the viral catastrophe entirely is difficult [52,53,54].

In our study, only SARS-CoV-2-positive patients were selected for sequencing the viral genome. During our whole study period, an increased frequency as well as a more virulent variant, Delta, was observed (June 2021 to December 2021), and at that time period, the aggressive and contagious Delta variant was dominant globally, where its wave began in May 2021 and continued to June 2021. In addition, in the later part of the study (January 2022–February 2022), a different mutational pattern, Omicron, was also reported, which was found globally at that time [55,56]. Socio-demographic and disease condition-related data of COVID-19 patients in Table 1 show that among the 25 COVID-positive patients, 48% (*n* = 12) were males, 52% (*n* = 13) were females, and none of them had chronic kidney disease, diabetes mellitus, chronic obstructive pulmonary disease, or a history of long-distance travel, whereas our previous research on COVID-19 conducted in a vast population reported that 47.20% and 3.27% of the COVID-19-positive patients had diabetes mellitus and chronic renal disease, respectively [57]. In this study, the sex distribution of COVID-19 contrasts our previous large-scale study comprising 8480 participants [57], which depicts those male subjects (42%) were prone to SARS-CoV-2 virus infection compared to females. In the analysis, it was found that people suffering from diabetes, chronic obstructive pulmonary disease, or chronic kidney disease before being infected with SARS-CoV-2 had poor disease progression, needed extra treatment, had a higher hospitalization rate, and had 4 times greater mortality rate if they were chronic obstructive pulmonary disease patients [58]. Among the study participants, 48% maintained a quarantine period, while 16% had a positive family history of COVID-19 infection. However, 12% of the subjects were reinfected with SARS-CoV-2, and 4% of them remained COVID-19 positive for a longer period of time, though 60% of them were vaccinated (Figure 1). Of most interest, 12% had hypertension, and 4% had asthma with hypertension, whereas our previous study revealed that 46.57% and 18.22% of COVID-19 patients had a history of hypertension and asthma, respectively [57]. Moreover, patients with asthma were reported to have severe disadvantages, with a higher risk of being infected, and suffered much more than the others [58].

Lineage and clade analysis of SARS-CoV-2 variants in Bangladesh demonstrated that 88% (*n* = 22) of the study subjects were carriers of Delta variants (Figure 2), whereas 20 of the 22 Delta variants fell in the 21A clade, and the other 2 fell in the 21J clade (Table 2). However, lineage evaluation via Pangolin revealed that the Delta variants evolved from nine lineages (i.e., AY.122, AY.122.1, AY.131, AY.26, AY.29, AY.30, AY.39, AY.4, B.1.617.2), where seven (28%) and six (24%) of the Delta variants were from B.1.617.2 and AY.4 lineages, respectively (Table 3). Moreover, two (8%) were from AY.131; the other two (8%) of the Delta variants were from the AY26 lineage; and the rest of the variants were from AY.122, AY.122.1, AY.29, AY.30, and AY.39 lineages, each comprising of 4% of all the 25 viral entities. In Ukraine, 25 lineages of the Delta variant were reported between February 2021 and January 2022, while in the case of our study, we found nine lineages, and among them, AY.122, AY.29, AY.4, and B.1.617.2 lineages were also found in the Ukrainian population [55]. The disappearance of one variant may be due to a lack of adaptability, while the emergence of the other can be demonstrated in the example of Delta B.1.617.1 and B.1.617.2 variants. In Maharashtra, these two variants comprised proportions of 55–60% and 10–60%, respectively, within the period of February to March 2021 [59]. However, in Bangladesh, the SARS-CoV-2 sample collected from July to August 2021 manifested that B.1.617.1 became rare in our population, while the second one (B.1.617.2) predominantly prevailed among the other eight lineages, comprising a proportion of 28%. However, the most prevalent clade of Omicron in different countries was claimed to be BA.1 among the three clades (i.e., BA.1.1, BA.2, and BA.3), which was also the same in the case of our observation in Bangladesh [60]. Hence, two (8%) of the Omicron variants follow the BA.1 lineage: one of them is from the 21K clade, and the other is from the 20A clade. After the emergence of Mauritius in January 2021, it spread at a high rate of eleven to seventy-five percent from March to May of that year [61]. However, in August 2021, Mauritius was identified in this present study at 4% (*n* = 1) incidence, which is lower than the estimated incidence in Gabon. The lineage of this variant in our country was identified to be B.1.318, which belongs to the 20B clade. However, in the present study, Delta, Mauritius, and Omicron were found, but not Alpha, Beta, and Gamma. This might be due to the spatial and temporal travel of variants of the SARS-CoV-2 virus.

In this cross-sectional descriptive study, the maximum number of substitution mutations in the Delta variant was 45, while the minimum number of this type of mutation was 14 (Table 4). On the contrary, the Delta variant that first emerged in India in October 2020 had 21 non-synonymous mutations and 5 synonymous mutations. Nevertheless, in place of only one deletion in the Indian Delta variant, the Delta variant from our country contains a maximum of four (creating a gap of 18 bp) and a minimum of three deletions (13 bp gap), which implies that at most 4× increased deletions occurred within this time frame of Delta traveling from India to Bangladesh (Table 4). Similar to the Indian Delta variant, the spike protein in this study also had a deletion at the 156/157 position (Table 11) [60]. Unlike the Indian variant, the Bangladeshi variant had an amino acid deletion at Y144 and R158 in S protein (Table 11). However, none of the Delta variants from our country was found to have an insertion mutation (Table 9).

The first emergence of Omicron (another name: GRA) was documented in November 2021 in several countries around the world. Though the number of mutations that Omicron carries is much higher than previous VOCs, it shares some common mutations with the previous variants. Notwithstanding, the first documented Omicron had 45 nonsynonymous mutations; in our genomic analysis, we found that it contains a maximum of 53 and a minimum of 41 substitution mutations (Table 4). Meanwhile, in place of seven deletions, we saw Bangladeshi Omicrons harboring a maximum of six (45 bp gap created) and a minimum of six (39 bp gap) deletion loci (Table 4) [60]. Additionally, two Omicron variants acquired 9 bp insertion mutation in the spike protein region (Table 4 and Table 9). In our study, Mauritius, on the other hand, was observed to have 36 substitution mutations, 5 (35 bp gap) deletion mutations, and 1 insertion (3 bp) mutation, where the number of mutations is higher than the previously recorded one (Table 4) [61,62,63].

The sequence of the viral genetic makeup was published by GenBank in January 2020 (accession No. MN908947.3). So far, the most frequent mutations of this viral genome are 23,403 A>G (D614G, in S protein), 241 C>T (in 5′ UTR region), 3037 C>T (in nsp3 of ORF1a), and 14,408 C>T (in nsp12 of ORF1b) [64,65]. However, in Bangladesh, the most commonly occurring mutations were detected to be 28,881 G>A (nucleocapsid protein (N) region), 23,403 A>G, 28,882 G>A (N), 28,883 G>C (N), 1163 A>T (ORF1a), and 14,408 C>T (ORF1ab) [5]. In our analysis, a total of 691 substitution mutations were observed, and among the identified mutations, A23403G, C3037T, C14408T, G15451A, C25469T, T26767C, C23604G, A28461G, and C21618G were the utmost frequent substitutions present in at least 20 (80%) of the 25 viral genomes (Table 5). D614G is one of the ten hotspot mutations that occurs at the frequency of 0.10 and was found to be associated with viral infectivity, greater transmissibility, and a higher fatality rate [66,67]. SARS-CoV-2 packaging and titers may be impaired due to mutations at non-coding regions like 5′ UTR, i.e., 241 C>T and 210 G>T [11,65]. Mutational spectra analysis of Asian and European SARS-CoV-2 demonstrated that 23,403 A>G, 3037 C>T, and 28,144 T>C (L84S, ORF8) mutations occur together with mutations 241 C>T, which have higher infectability [68]. Similarly, in the present study, a co-occurrence of 241 C>T with 23,403 A>G, 3037 C>T, and 28,144 T>C mutations was seen. It was noticeable that 3037 C>T and 23,403 A>G and 14,408 C>T mutations were seen in most of the cases (Table 5). Moreover, mutation 241 C>T tends to coincide with mutation 210 G>T most of the time among these twenty-five adult COVID-19 Bangladeshi patients, which should be underscored to further evaluate the molecular and biological aspects of viral behavior.

It was reported that host cellular enzymes modify host RNAs as well as viral RNAs, like the methylation of adenosine and deamination (by ADAR and APOBEC enzymes) of cytidine to uracil and adenosine to inosine. Deamination results in C to T and A to G change in the sequence, which explains the fact that C-T and A-G mismatch occurs during multiple sequence alignment with the wild-type reference genome of SARS-CoV-2. As a result, the deamination of cytidine and adenosine was held accountable for 65% of the C>T synonymous substitutions as well as 22% of the A>G substitution mutations [40,69]. In our study, we also found that the frequency of C>T (38.48%) mutation was much higher than that of G>T (18.23%) and A>G (11.52%) mutation (Table 6). It is also mention-worthy that, so far, in our study, we observed that transition (T>C or G>A) mutations occur more often than transversion mutations (i.e., T>G or A>C), and the percentages of transition as well as transversion mutations were 67% and 33%, accordingly (Table 7). As mentioned earlier, the possible reason behind this could be the increased rate of deamination of cytidine to uracil and adenosine to inosine. However, further studies can be conducted to explain this phenomenon as well.

As presented in Table 8, deletions observed in the Delta variant genome include 22,029–22,035 (S), 28,248–28,254 (ORF8), and 28,274 (N), as well as 29,750–29,752 (non-coding) in the case of the 21A clade and 21,992–21,994 (S), 22,029–22,034 (S), 28,248–28,253 (ORF8), and 28,271 (non-coding) in the case of the 21J clade, which resides in the spike (S) protein-encoding gene, in the nucleocapsid-encoding gene, and in ORF8, as well as in a non-coding region. Research suggests that deletion at the S gene can pose many more challenges because it can upsurge the ability of spike protein attachment with the ACE2 protein of the host cell and therefore increase transmissibility or even pathogenicity [59]. All the aforementioned deletions of the 21J clade were present in both of the variants except the deletion at 21,992–21,994, which was seen only in a sample (accession number OM277498), which gives a hint that the latter one may gather more advantageous mutations and evolve as a new variant of interest. On the other hand, most of the deletions in the Omicron genome were unique and were not found in Delta or Mauritius variants. Moreover, the Omicron variant’s 20A clade contains deletions at six loci, i.e., 6513–6516 (ORF1a), 11,287–11,296 (ORF1a), 21,766–21,772 (S), 21,987–21,996 (S), 22,194–22,197 (S), and 28,363–28,372 (N), and among them, deletion at two loci of ORF1a was not seen in the Delta variants, though deletion at around the 11.3 kbp site was seen in the Mauritius variant. However, though at around the same loci, the 21K clade of the Omicron variant contained a slightly different range of deletion, varying in 1 bp or 2 bp, and they include 6513–6515 (ORF1a), 11,285–11,293 (ORF1a), 21,765–21,770 (S), 21,987–21,995 (S), 22,194–22,196 (S), and 28,362–28,370 (N) deletions residing in ORF1a, the spike glycoprotein, and nucleocapsid gene regions. Deletion at the S gene site may facilitate viral transmission, infectivity, pathogenicity, and/or vaccine efficacy. However, the Mauritius variant includes 11,288–11,297 (ORF1a), 21,994–21,997 (S), 27,887–27,902 (ORF7b, ORF8), 28,254 (ORF8), and 28,896–28,899 (N) deletion mutations, which also have unique deletions at ORF7b and ORF1a sites, along with deletions at S, N, and ORF8 genes. To sum up, the Omicron variant has more diverse deletions at vulnerable sites of the S gene than the Mauritius variant and the lowest number of deletions encompassed by the Delta variant.

While looking for insertion mutations in our investigation, we found that two insertion mutations of 9 bp length in the spike glycoprotein region were seen in two of the Omicron variants, 22206: GCCAGAAGA (in 20A clade) and 22204: GAGCCAGAA (in the 21K clade) (Table 9). In the same time period between February 2021 and January 2022, in Ukraine, three in-frame insertion mutations were reported in 62 Omicron variant samples [55]. In the case of the Mauritius variant, a 3 bp (28250: CTG) insertion mutation was observed in the ORF8 region.

To gain a bird’s eye view of the mutational spectra of S and N proteins and OR1a and ORF1b polyproteins, lollipop plots of amino acid substitution (red lollipop) and deletion (blue lollipop) mutations in all of the three variants are given in Figure 3, Figure 4, Figure 5 and Figure 6, accordingly. In Figure 3, it is clear that for the most significant one, the spike protein of the Omicron variant, most of the unique mutations, as well as deletion and substitution mutations, were noticed. Substitution mutations T19R, T95I, E156G, L452R, T478K, D614G, P681R, and D950N were observed in most of the Delta variants, and T95I, as well as D614G, was found in all three variants. Moreover, P681 was substituted with R (Arginine) in Delta but with H (Histidine) in Omicron and Mauritius variants, while A67 was substituted with V (Valine) in Omicron but substituted with T (threonine) in the Mauritius variant, which refers to a phylogenetic deviation point. In addition, D796 was substituted with Y (Tyrosine) in Omicron and with H in the Mauritius variant, while E484 was substituted with Q (Glutamine), A (Alanine), and K (Lysine) in the Delta, Omicron, and Mauritius variants, respectively. Furthermore, G142D and H655Y were found both in Delta and Omicron variants. Moreover, in the case of the Omicron variant, most of the substitution mutations were concentrated in the Receptor Binding Domain (RBD), while deletion mutations were found to be clustered in the N-terminal region of the S1 functional subunit of the spike glycoprotein. In the time frame of February 2021-January 2022, the main high-frequency mutations in the S protein of the VOC Delta variant were reported to be T19R, G142D, E156G, F157del, R158del, L452R, T478K, D614G, P681R, and D950 N [55]. However, in our case, we found G446V and N501T missense mutations in high frequency in the spike protein. In the case of the Omicron variant, in Ukraine, D614G, D796Y, G339D, H655Y, N679K, N764K, N969K, P681H, Q954H, S373P, K417 N, T478K, N440K, S375F, G142D, S477 N, Q493R, Q498R, N501Y, E484A, A67V, G446S, L212I, L981F, N856K, S371L, T547K, G496S, and T95I missense mutations were observed in the S protein, which were collected from February 2021 to January 2022 [55], and all these mutations were also found in the case of our study samples of Omicron variants.

However, in the nucleocapsid protein (N) of the Delta variant, no amino acid deletion was observed, and as we have seen in the nucleotide deletion section, only a single bp was deleted at the 28,274 loci, which eventually did not result in AA deletion. In addition, compared to the other two variants, most of the mutations were in the Delta variant (Figure 4). Interestingly, only R203 was found to be substituted in all these three variants, but it was substituted to M (Methionine) in Delta, while it was substituted to K (Lysine) in Omicron and Mauritius variants. In the Omicron and Mauritius variants, the common mutation was G204R. Moreover, M1X, D63G, R203M, G215C, and D377Y were the most frequent mutations in Delta variant samples, and in Ukraine, the N protein of the Delta variants during February 2021–January 2022 was reported to have these mutations in high frequency, except M1X [55]. Moreover, deletion at the E31del, R32del, and S33del positions of the N protein was reported in the Omicron variant in Ukraine during the same time period [55].

Regarding ORF1a, in the case of Delta mutations, it encompassed the whole polyprotein region, and the other two variants showed fewer mutations in the N terminal regions, from which angle we can consider further research aimed at figuring out their changes in viral behavior (Figure 5). In Delta variants, K261N, P309L, A1306S, P1640L, H2092Y, P2287S, V2930L, A3209V, T3255I, T3646A, and V3718A were the most frequent mutations. Moreover, when comparing the variants, only L3606F was observed to be common in all three variants, and A3209V was observed in the Delta and Mauritius variants, whereas T3255I was found in both Delta and Omicron variants. However, though S3675-, G3676-, and E3677- deletion mutations were found to happen in the Omicron and Mauritius variants, no deletion mutation was found in the Delta variant’s ORF1a polyprotein.

In our investigation, it was revealed that the ORF1b protein did not have mutations at all after the 2473th amino acid residue, in other words, downright from the N terminal domain of NSP4 in all these three variants (Figure 6). In the case of the Delta variants, P314L, G662S, P1000L, and A1918V were the most frequent mutations in the viral genomes. When comparing the variants of Delta, Omicron, and Mauritius, it was observed that P314L was present in all three variants, and in some samples of the Delta variant, in place of L (Leu), P314 was substituted with F (Phenylalanine), which might indicate the emergence of a new strain of the Delta variant. Moreover, V2371 was substituted with L (Leu) in the Delta variant, which was substituted with M (Methionine) in the Mauritius variant, indicating different substitutions, raising new research interest in the question of whether they can yield more advantages, i.e., post-translational modification to the latter [70]. In a nutshell, it can be said that the mutational spectrum analysis provides clear insights into the significant mutational variations and gradual changes of the viral genome until it evolves into a new strain or variant of interest.

Recent research implies that the unique and characteristic spike protein mutations of Delta variants include P681R, T19R, T478K, L452R, D614G, and D950N, as well as deletion at the part of the N-terminal domain region, Δ157–158. Higher transmissibility of the Delta variant can be demonstrated by the adoption of 6 point mutations in spike protein gene over time, and the noteworthy among them are P681R and L452R, as well as the aforementioned deletion at Δ157–158, residing in the Receptor Binding Domain, and altogether, they were found to be associated with an increase in binding to the ACE2 protein and antibody neutralization to evade the immune system [59]. The point mutation at the S1–S2 cleavage site of spike protein P681R found in Delta variants in our study (Figure 3a) yields an assumption that this deletion might be associated with its strain’s higher replication ability, resulting in higher viral load and increasing transmission [71]. In our investigation, 30 signature/unique mutations were found in the spike protein of Omicron variants among the twenty-five adult COVID-19 patients (Figure 3). Due to the various unique deleterious alterations, especially in the Receptor Binding Domain (RBD) of the spike glycoprotein, where in place of 4 unique alterations detected in the Delta variant, Omicron contains 12 unique variants that are absent in Delta (Figure 3a,b), it is believed that the Omicron variant intensified health concerns by posing an increased risk of reinfection, reducing the treatment response (which threatens the treatment strategy) and increasing host immunity evasion [72,73,74].

Regarding the Mauritius variant, Cherian et al. hypothesized that this variant possesses some unique mutations that are common in this clade, like P618R, L452R, and E484Q in the S gene region, which might be associated with the rapid infections and transmission in the province of Eastern Maharashtra [59]. Similarly, in this present study, Y144-, D614G, D796H, E484K, P681H, and T95I were identified in the spike protein of the Mauritius variant (Figure 3c). Nevertheless, in the Republic of Gabon, this variant was reported as carrying P681H, E484K, D614G, D796H, and T95I, as well as deletion at Y144-, where they proposed that D614G might have a role in the rapid transmission of this VOI. Furthermore, it was stated that E484K and Y144- have an impact in decreasing vaccine efficacy.

It was documented that the genes of the ORF1ab open reading frame, i.e., *nsp1*-*3*, *nsp12*, and *nsp15*, as well as the spike protein gene, *S*, and *ORF8* gene, were reported to have remarkably more mutations than other genes [40]. On the other hand, in the present study, ORF1a, ORF1b, ORF3a, and nucleocapsid genes were detected to acquire a comparatively significant number of substitution mutations than the other genes (Figure 7). Moreover, an analysis of deletion mutations unraveled that most of the aa deletion mutation was acquired by the variants at S and ORF8 genes, while no aa deletion mutation was observed in ORF1b, ORF3a, E, M, ORF7a, and ORF7b proteins (Figure 7).

To conclude, it can be said that the results presented here in this investigation can provide new insights into the mutational spectra of SARS-CoV-2 viral genome and can help in constructing a genetic database of COVID-19 of Bangladeshi adult COVID-19 patients based on the course of the period from the very beginning of this disease incidence in Bangladesh till this date, and genetic surveillance should be continued as this SARS-CoV-2 has yet not been eradicated from our country or many parts of this world. The remarkable and significant findings of our investigation on this novel viral genome shaded light on the demand for understanding the molecular basis of SARS-CoV-2 genetics isolated from Bangladeshi COVID-19 patients and might have value in the near future in the field of the diagnosis of the disease in medicinal treatment, the development of vaccines, and decision making in its management. Moreover, by exploring the evolution of mutational spectra of SARS-CoV-2 variants of concern or variants of interest from different global regions at different time frames, researchers can gain an understanding of the preference of the virus’s genetic mutation types and preference for mutation sites that the virus tends to acquire to increase its adaptation strategy, transmissibility, survival, pathogenicity, and resistance against drugs, which ultimately leads to the emergence of newly evolved viral strains with extraordinary advantages over the old virus. Moreover, the findings of the study on the viral genome can add a new dimension to unraveling the challenges that SARS-CoV-2 imposed.

## Figures and Tables

**Table 1:** Socio-demographic and comorbidity of the COVID-19 patients.

Socio-Demographic Variables and Comorbidity	Number	Percentage (%)
Male	12	48
Female	13	52
Family history of COVID-19 infection	4	16
Maintained quarantine period	12	48
Hypertension	3	12
Hypertension with asthma	1	4
Re-infected with SARS-CoV-2	3	12
Vaccinated	15	60
Long duration of COVID-19-positive	1	4

**Table 2:** Frequency of identified clades of SARS-CoV-2.

Variant	Clade	Number	Percentage (%)
Delta	21A	20	80
	21J	2	8
Omicron	20A	1	4
	21K	1	4
Mauritius	20B	1	4

**Table 3:** Lineage distribution of SARS-CoV-2 variants.

Variant	Lineage	Number	Percentage (%)
Delta	B.1.617.2	7	28
	AY.4	6	24
	AY.131	2	8
	AY.26	2	8
	AY.29	1	4
	AY.30	1	4
	AY.39	1	4
	AY.122	1	4
	AY.122.1	1	4
Omicron	BA.1	2	8
Mauritius	B.1.1.318	1	4

**Table 4:** The types of mutations (in numbers) observed in each COVID-19 patient.

Mutation	Variant				
	Omicron		Delta		Mauritius
	Maximum	Minimum	Maximum	Minimum	
Substitution	53	41	45	14	36
Deletion sites with gap in bp	6 (45)	6 (39)	4 (18)	3(13)	5 (35)
Insertion sites with length in bp	1 (9)	0	-	-	1 (3)

**Table 5:** List of substitution mutations with their mapped gene/genomic regions in SARS-CoV-2.

Genomic Region	Substitution	Number of Mutations	Genomic Region	Substitution	Number of Mutations
ORF1a	C3037T	25	S	C23604G	22
	C10029T	16		C21618G	20
	G4181T	15		G24410A	17
	C6402T	15		C22995A	16
	C8986T	15		T22917G	14
	G9053T	15		C21846T	9
	A11201G	14		G21987A	5
	A11332G	14		G22899T	5
	C7124T	10		A23064C	4
	C9891T	7	ORF3a	C25469T	22
	C5184T	6		G26104T	4
	T12946C	6	M	T26767C	23
	C1191T	5	ORF7a	C27752T	14
	C1267T	5		T27638C	13
	T11418C	5	ORF7b	C27874T	15
	G1048T	4	ORF8	C28054G	5
	A2560G	4	N	A28461G	21
	G11083T	4		G28881T	19
ORF1b	C14408T	25		G29402T	15
	G15451A	22		G28916T	14
	C16466T	19		T29014C	4
	C19220T	15	5′ Leader Sequence	C241T	16
	C14407T	5		G210T	14
	G19999T	5	near 3′ end	G29742T	14
S	A23403G	25		G29688T	5

**Table 6:** Nucleotide base changes of substitution mutations in the SARS-CoV-2 viral genome.

Nucleotide Base Change	Number	Percentage (%)
C>T	344	38.48
G>T	163	18.23
A>G	103	11.52
G>A	77	8.61
T>C	75	8.39
C>G	48	5.37
C>A	33	3.69
T>G	22	2.46
A>C	10	1.12
A>T	7	0.78
G>C	6	0.67
T>A	6	0.67

**Table 7:** Comparison of frequency of transition (i.e., T>C, G>A) and transversion (i.e., T>A, G>C) mutation in SARS-CoV-2.

Mutation	Base Change	Number	Percentage (%)
Transition	Purine > Purine/ Pyrimidine > Pyrimidine	599	67
Transversion	Purine > Pyrimidine/ Pyrimidine > Purine	295	33

**Table 8:** Deletion mutations in the SARS-CoV-2 viral genome from distinctive clades, along with their genomic annotation.

Deletion (bp) of Variant (Gene)				
Delta		Omicron		Mauritius
21A	21J	20A	21K	20B
22,029–22,035 (S)	21,992–21,994 (S)	6513–6516 (ORF1a)	6513–6515 (ORF1a)	11,288–11,297 (ORF1a)
28,248–28,254 (ORF8)	22,029–22,034 (S)	11,287–11,296 (ORF1a)	11,285–11,293 (ORF1a)	21,994–21,997 (S)
28,274 (N)	28,248–28,253 (ORF8)	21,766–21,772 (S)	21,765–21,770 (S)	27,887–27,902 (ORF7b, ORF8)
29,750–29,752 (Non-coding)	28,271 (Non-coding)	21,987–21,996 (S)	21,987–21,995 (S)	28,254 (ORF8)
		22,194–22,197 (S)	22,194–22,196 (S)	28,896–28,899 (N)
		28,363–28,372 (N)	28,362–28,370 (N)	

**Table 9:** Insertion mutation and their genomic locations in different types of SARS-CoV-2 viral genomes.

Variant (Clade)	Insertion Position with Inserted Sequence	Length (bp)	Mapped Gene
Omicron (21K)	22,204: GAGCCAGAA	9	S
Omicron (20A)	22,206: GCCAGAAGA	9	S
Mauritius (20B)	28,250: CTG	3	ORF8

**Table 10:** Amino acid substitution in SARS-CoV-2 viral genome.

Genomic Region	Amino Acid Substitutions	Number of Mutations	Genomic Region	Amino Acid Substitutions	Number of Mutations
ORF1a	T3255I	16	S	D950N	17
	A1306S	15		T478K	16
	P2046L	15		L452R	14
	V2930L	15		T95I	9
	T3646A	14		G142D	6
	P2287S	10		G446V	5
	A3209V	7		N501T	4
	P1640L	5		H655Y	3
	P309L	5		P681H	3
	V3718A	5	ORF3a	S26L	22
	K261N	4		D238Y	4
	L3606F	4	M	I82T	24
	H2092Y	3	ORF7a	T120I	14
ORF1b	G662S	22		V82A	13
	P314L	20		L116F	3
	P1000L	19	ORF7b	T40I	15
	A1918V	15	ORF8	S54 *	5
	P314F	5	ORF9b	T60A	21
	V2178F	5	N	M1X	20
S	D614G	25		D63G	19
	P681R	22		R203M	19
	E156G	20		D377Y	15
	T19R	20		G215C	14

*—stop codon.

**Table 11:** Amino acid deletion mutations of SARS-CoV-2 viral genome.

Gene	Deletion Mutations	Number of Mutations
ORF1a	S3675-	3
	G3676-	3
	F3677-	2
	L2084-	1
	S2083-	1
	L3674-	1
S	F157-	22
	R158-	20
	E156-	2
	H69-	2
	V70-	2
	V143-	2
	Y144-	4
	Y145-	1
	L212-	1
	G142-	1
	N211-	1
ORF8	D119-	22
	F120-	22
	M1-	1
	K2-	1
ORF9b	N28-	2
	A29-	2
	V30-	1
	E27-	1
N	E31-	2
	R32-	2
	S33-	2
	R209-	1

## Data Availability

All the data are accessible using the https://www.ncbi.nlm.nih.gov/ website via the following accession numbers: OM019149, OM019148, OM019138, OM019145, OM019139, OM019150, OM019143, OM019146, OM090130, OM090137, OM090136, OM090135, OM090140, OM019153, OM019152, OM019154, OM019140, OM019147, OM019141, OM019155, OM090139, OM277491, OM277497, OM277498, and OM019151. 10.6084/m9.figshare.27263484. The original data presented in this study are openly available in FigShare at https://figshare.com/arti- cles/dataset/Metadata/27290031, accessed on 17 December 2024.

## Supplementary Materials

The following supporting information can be downloaded at: https://figshare.com/articles/dataset/Metadata/27290031 (accessed on 17 December 2024). Substitution and amino acid substitution mutations are presented in the Supplementary Materials. Table S1. Nucleotide substitution mutations in three different SARS-CoV-2 variants enlisted with their genomic annotations observed in three samples at most. Table S2. List of amino acid substitution mutations and annotated genes with their number of occurrences, which were found in the fewest number of samples.
